# *Bunch Ash* biomass source for the synthesis of Al_2_(SiO_4_)_2_ magnetic nanocatalyst and as alkali catalyst for the synthesis of biodiesel production

**DOI:** 10.1016/j.mex.2023.102304

**Published:** 2023-08-01

**Authors:** E.R. Akhabue, S.E. Onoji, F. Ishola, A.A. Ukpong, O. Idama, U. Ekanem, T.F. Adepoju

**Affiliations:** a Department of Chemical Engineering, Faculty of Engineering & Informatics, University of Bradford, United Kingdom; b Petroleum and Natural Gas Processing Department, Petroleum Training Institute, Effurun, Delta State, Nigeria; c Southern Alberta Institute of Technology, SAIT, Calgary, Canada; d Chemical/Petrochemical Engineering Department, Akwa-Ibom State University, Ikot Akpaden, Akwa-Ibom State, Nigeria; e Department of Computer Engineering, Delta State University of Science and Technology, Ozoro, Delta State, Nigeria; f Chemical Engineering Department, Delta State University of Science and Technology, Ozoro, Delta State, Nigeria

**Keywords:** Admixture, API gravity, Biodiesel, Process optimization, Response surface methodology, Catalyst characterization, Physiochemical properties, Recyclability test, API gravity ratio, Transterification, Process Optimization-RSM

## Abstract

This work employed the Admixture of oil from winter squash seed oil and duck waste fat for the synthesis of biodiesel using a derived heterogeneous catalyst from burnt *Arecaceae* kernel empty bunch (BAKEB). The admixture oil was obtained using the gravity ratio method and the properties of the oils were determined. The developed BAKEB was characterized using SEM, FTIR, XRF-FT, BET-adsorption, and qualitative analysis. Transesterification of the admixture oil to biodiesel was carried out in a single transesterification batch reactor, while Process optimization was carried out via RSM-CCD with four constraint variables namely: reaction period, catalyst conc., reaction temperature, and E-OH/OMR, respectively. The spent catalyst was recycled and reused and the quality of the produced biodiesel was compared with the recommended standard. Results showed the admixture oil ratio of 48:52 was sufficient to produce a validated optimum biodiesel yield of 99.42% (wt./wt.) at the reaction time of 55 min, catalyst conc. of 3.00 (%wt.), reaction temperature of 60 °C, and E-OH/OMR of 5.5:1 (vol./vol.), respectively. ANOVA analysis indicated that all variables were mutually significant at p-value<0.0001.The developed BAKEB was found to contain high percentages of Al-K-Na-Ca. The catalyst recyclability test indicated that BAKEB can be refined and reused. The produced biodiesel qualities have fuel properties similar to conventional diesel when compared with ASTM D6751 and EN 14,214. The study concluded that the blending of winter squash seed oil with duck waste fat in the ratio of 48:52 as feedstock for biodiesel synthesis is viable.

## Review Highlights

• Low viscous oil was obtained via the admixture of winter squash seed oil and duck waste fat.
• The catalyst derived from burnt *Arecaceae* kernel empty bunch (AKEB) contained high K-Al-Ca.
• Biodiesel properties are in conformity with recommended biodiesel standard.
• A single stage transesterification batch reactor was employed.

## Graphical abstract

**Figure:**
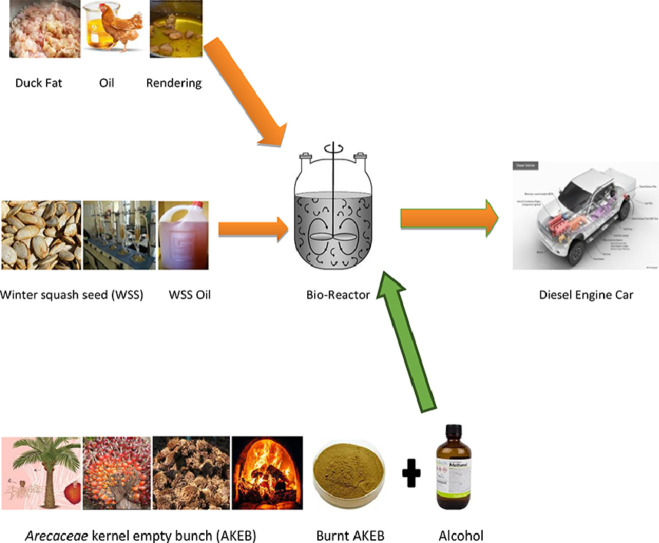

Specifications table

**Table:** 

Subject area:	Energy
More specific subject area:	Biofuel
Name of the reviewed methodology:	API gravity ratio, Transterification, Process Optimization-RSM
Keywords:	Admixture, API gravity, Biodiesel, Process optimization, Response Surface Methodology, Catalyst characterization, Physiochemical properties, Recyclability test
Resource availability:	Three-necked-reactor, SEM, FTIR, XRD, BET analyzers, grinder, design expert 360 stat ease, viscometer, density bottle, iodine tester, continuous extractor, rotatory evaporator, hot plate with magnetic stirrer.
Review question:	How do we admixture two or more oil? Does transesterified oil require low viscous properties? Does *Arecaceae* kernel empty bunch possess alkali properties? What are the variables required for successful biodiesel conversion? Can RSM be used for process optimization for optimum yield validation? Is the developed alkali recyclable and reusable? Can the biodiesel produced be used as replacement for conventional diesel?

## Introduction

Presently, based on world meters information, the world population clock 8 billion in 2023. The world's energy consumption has continuously grown over the past half decay, reaching 25,300 terawatt-hours (Statistical Review of World Energy, 2021). Meanwhile, the energy supply and consumption which is a universal creation and provision of fuel, generation of electricity, energy transport, and energy consumption have been estimated at 14,500 terawatt-hours [1]. This showed that the supply is less than the demand which calls for urgent attention for the economic growth of any nation. Furthermore, greenhouse gas emissions estimated at 50 billion have been reported to occur as a result of fossil fuels causing more havoc to climatic change that affects the human race [2,3]. The goal set in the Paris Agreement to limit climatic change that occurred as a result of fossil fuel using several scenarios has not been reached, yet the stratospheric ozone depletion keeps on growing. The only way to salvage the world from exposure to the overly increasing pandemics caused by the ozonized-depletion is to invest in cleaner burning of gasoline. This is indeed difficult to achieve due to cost implications. However, the use of fuel generated from biomass feedstock has been reportedly eliminated the emission problems, providing a lasting solution to additional problems such as scarcity of fuel, non-renewability, and high cost of transportation [4,5]. This fuel also known as Biofuel such as biodiesel produced via transesterification or esterification before transesterification has been obtained from vegetable oil, algae, and fat oils [6,7].

Winter squash is the annual fruit of the species-genus *Cucurbita*. Odd-shaped, warty, small to medium in size, but with hard rinds. They are harvested and eaten in the mature stage when their seeds within have matured fully and with hardened tough rind. The seeds are edible and nutritious when roasted like butternut, contain protein, and fiber, and are healthier than traditional snack with a capacity to reduce cancer, diabetes, arthritis, and respiratory disease. Nevertheless, the matured seed when extracted via Soxhlet extraction has been reported to contain a low acid value with a 35.67% yield [8].

The transesterification process involved the use of catalysis for the complete conversion of oils to biodiesel. The common alkali base catalysts being employed so far are sodium hydroxide (NaOH), potassium hydroxide (KOH), potassium methoxide (CH3OK), and sodium methoxide (CH3ONa) [9]. However, the use of calcium/potassium based derived from solid wastes is preferable over the use owing to thigh activity and selectivity, green catalysts, less energy consumption, recyclability, high product formation, and minimum time needed to achieve the desired product. Meanwhile, admixture or blending or mixing of oils has been reportedly helped to increase the yield of the product while lowering the viscous effects of the blended oil [[10], [11], [12]].

Furthermore, different software have been used for modeling and optimization of biodiesel production such as response surface methodology (RSM) [13,14] genetic algorithm (ANN) [15], [16], [17], [18], Neuro fussy intelligent system (NFIS) [19], [20], [21], [22], [23], MCDM analytical hierarchy [18,19], Tanquchi L9, Python [13] and many more. This software assists researchers to evaluate the predicted yield, determining the analysis of variance, evaluating the test of significant, determining coefficients of determination, modeling the polynomial model equation, enumerating the optimum product yield, and minimizing the conditions of the variables (catalyst concentration, reaction time, reaction temperature, reaction speed, and alcohol to oil molar ratio).

Thus, this research study employed the admixture of winter squash seed oil with duck fat in the presence of a derived catalyst for biodiesel synthesis. The derived catalyst was obtained from burnt *Arecaceae* kernel empty bunches and characterized using Fourier transform infrared spectroscopy (FT-IR), energy dispersive X-ray fluorescence (XRF) spectroscopy, scanning electron microscope (SEM), BET surface area measurements, and CO2 temperature programmed desorption (CO2-TPD). A single-stage batch reaction was employed for a low acid transesterification of oil to biodiesel. The resultant biodiesel quality was examined via the physical, chemical, and fuel properties determinations and the results were compared with the recommended biodiesel standard. Finally, the strength of the catalyst was tested by recycling, refining, and reusability.

## Experimental

A continuous extractor was employed for the extraction of oil from the powder seed of winter squash using an analytical solvent (n-hexane) [8]. To a 500 mL of continuous extractor, 50 g of the powder was loaded in the thimble, and 300 mL of n-hexane was measured into the round bottom conical flask placed in the water bath, and the reaction was monitored for 120 min until the oil was completely leached out of the powder seed at 72 °C temperature. The extracted oil with excess n-hexane was recycled using a rotary evaporator, and the oil was filtered using a filtration unit to obtain clean oil. These processes were repeated severally until 5-L of the oil was produced and stored for further processing.

### Rendering of poultry fat

Duck fat was cut into small sizes of 2 inches in extractors and the extractor was heated at 120 °C until the fat completely melted with the stirring speed kept at 250 rpm to maintain a homogeneous oil phase [23]. The rendered oil was allowed to cool at room temperature and then filtered to eliminate the residual fat meat. The cleaned oil was stored for blending/admixture.

### Admixture oil

Admixture of the oil was carried out based on the API gravity determination of the individual oil. The API gravity of the oil was first determined; the API gravity total was then obtained by summing up the individual API gravity of each oil [3]. The percentage admixture ratio was computed using Eqn. (1), and this was used for the oil Admixture ratio to obtain low viscous oil used as a raw material for the synthesis of biodiesel.

(1) $$\text{Admixture}\;\text{ratio}=\left(\frac{\tau_{WSO}}{\tau_{WSO+DFO}}:\frac{\tau_{DFO}}{\tau_{WSO+DFO}}\right)$$

Where: $\tau_{WSO}$ is the API gravity of the winter squash oil; $\tau_{DFO}$ is the API gravity of the duck fat oil; $\tau_{WSO+DFO}$ is the total API gravities of both oils.

## *Properties of admixture oil*

Properties such as density, viscosity, moisture content, acid value along with FFA, peroxide, saponification, iodine values, and other parameters were evaluated following the standard procedures from AOAC, 1997, to determine its suitability for biodiesel production. Table 1 shows the results of the properties of the winter squash oil (WSO), the duck fat oil (DFO), and the admixture oil.

**Table 1:** **Properties of oils**.

Properties	WSO	DFO	Admixture oil
Density (kg/m^3^) @ 30 °C	770.00	860.00	866.00
Viscosity @ 40 °C/ (mm^2^/s)	14.30	13.60	13.86
Moisture content (%)	0.001	0.001	0.001
%FFA (as oleic acid)	0.60	0.67	0.63
Acid value (mg KOH/g oil)	1.20	1.34	1.26
Saponification value (mg KOH/g oil)	195.40	190.60	192.20
Iodine value (g I_2_/100 g oil)	87.60	82.40	82.00
Peroxide value (meq O_2_/kg oil)	6.50	5.70	5.46
HHV (MJ/kg)	40.11	40.37	40.31
Cetane number	54.51	56.41	56.26
API gravity	29.29	31.14	29.29
Blend ratio	48	52	–

API gravity of oil >10, lighter oil, API gravity < 10, heavier oil.

### *Catalyst analysis and characterization*

**Fig. 1:**
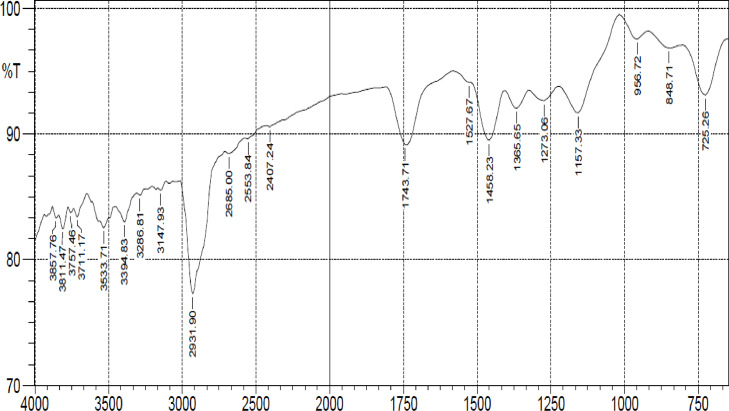
FTIR pictorial view showing the peaks.

**Fig. 3:**
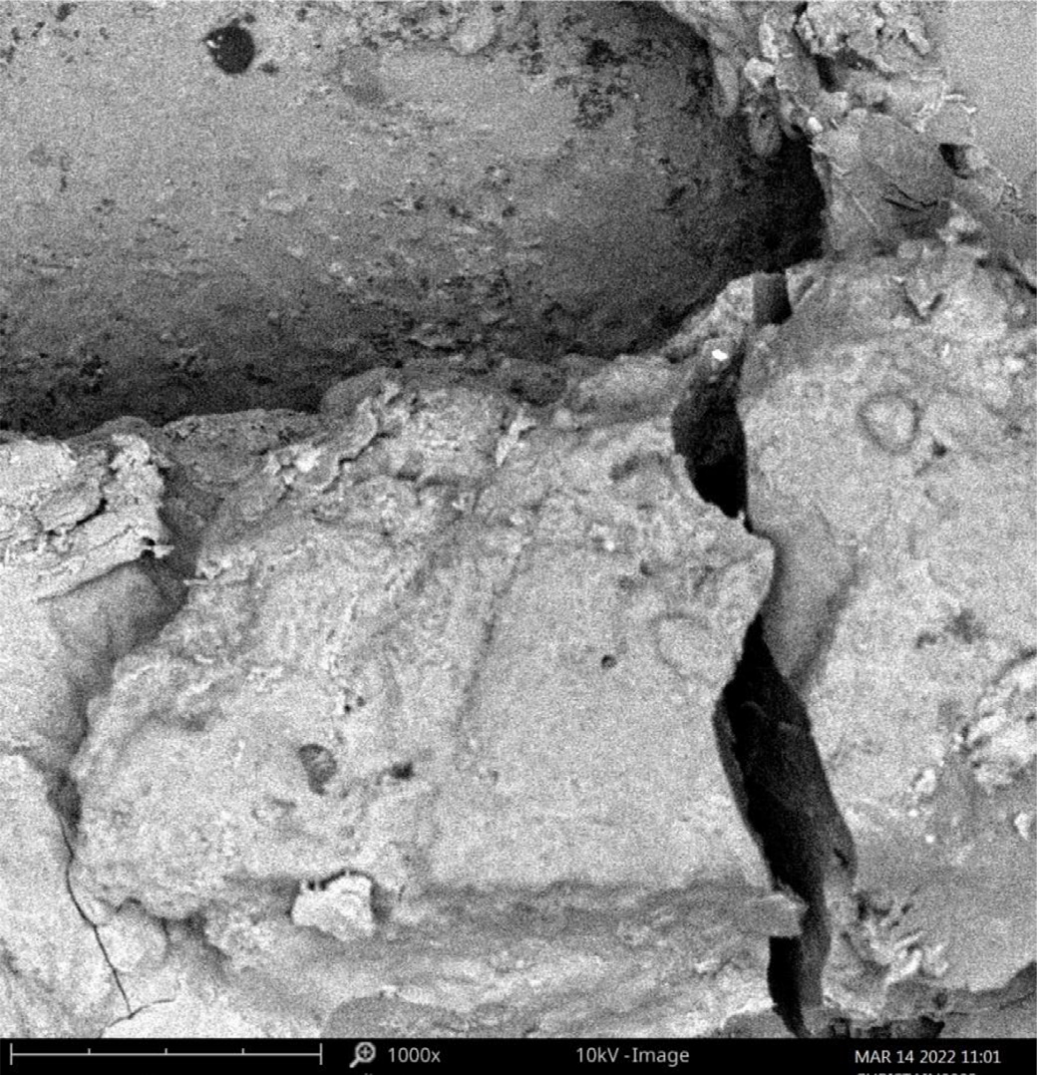
SEM analysis structure.

**Fig. 4:**
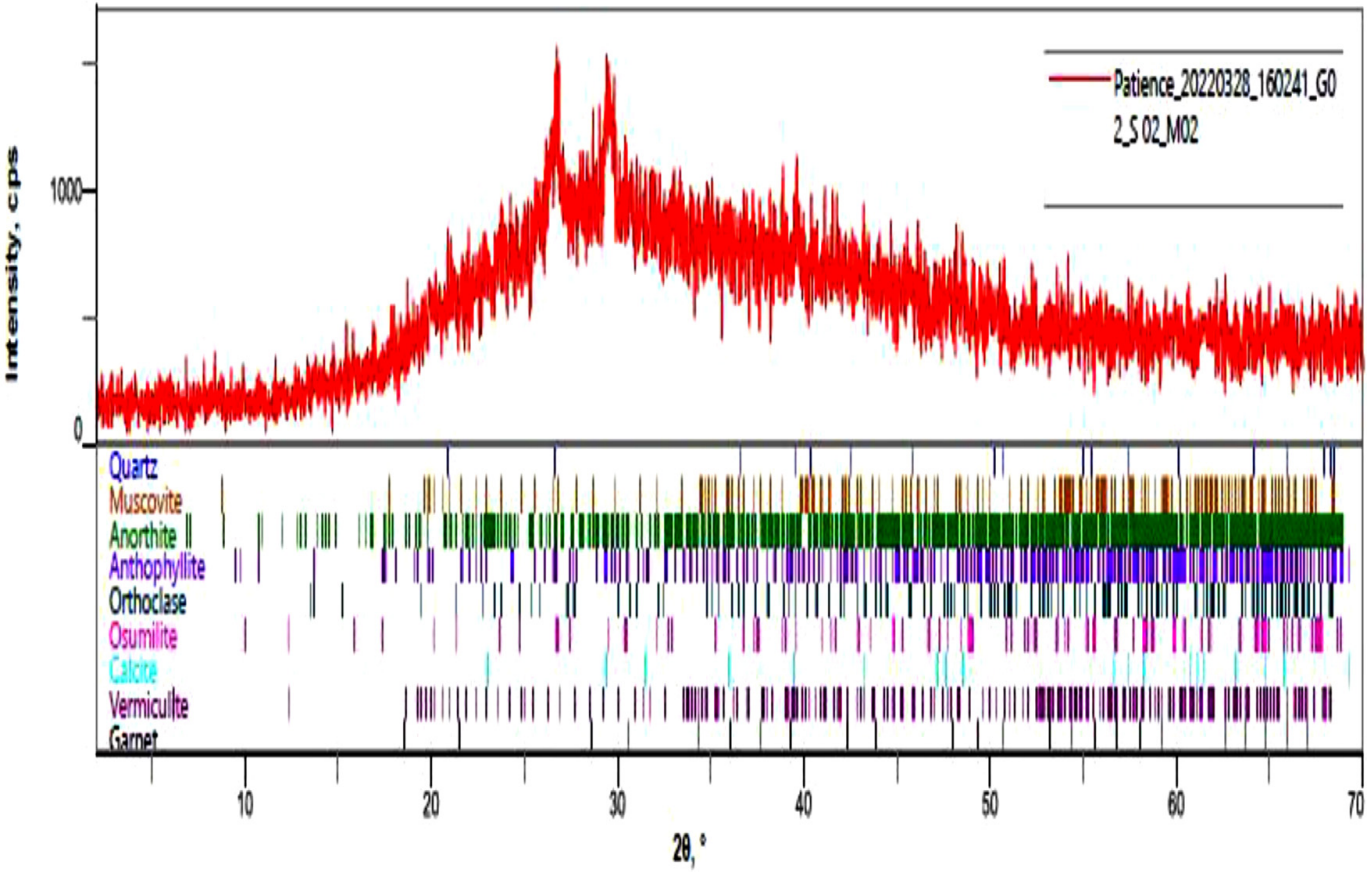
Qualitative analysis with structural compound.

The burnt catalyst was made into a fine powder and was characterized as earlier mentioned. Fig. 1 shows the FTIR pictorial view indicating the peaks of elements and functional groups present in the derived catalyst. Fig. 2 displayed the SEM image of the analyzed catalyst characterized at a magnification of 1000x. The image shows the jointly-cracked structure with a whitish ash-like, soapy nature, which indicated the presence of white metals ranging from white, silvery white to dull gray. The sintering of small mineral aggregates and agglomerated particles responsible for the squishy nature of the catalysts can be traced to thermal heat treatment. This is an indication that the heat treatment resulted in the spontaneous release of oxide of calcium (CaO) during the burning of the bunch kernel which liberated carbon dioxide (CO_2_) from calcium carbonate (CaCO_3_) and Aluminium from the oxide. Other elemental compounds were also present in a small proportion that aids the transesterification of oil to biodiesel. Displayed in Fig. 4 is the qualitative analysis view of the catalyst. The structural displays the compounds present in the catalyst indicate the presence of the alkalis compounds (Fig. 3).

Table 2 presented the functional compounds found in the derived catalyst during XRF-FS analysis. The compounds indicated the presence of calcium, potassium, aluminum, silicon, magnesium, and other elements that helps during the transesterification conversion of oil to biodiesel. The BET adsorption analysis indicating the performance of Langmuir and Isotherm based on data reduction is also presented in Table 3. This determines the surface area, the pore volume, and the total basic density of the catalyst.

**Table 2:** XRF-FS analysis results.

Name	Compound
Quartz	SiO_2_
Muscovite	KAl_2_(Si_3_Al)O_10_(OH, F)_2_
Anorhite	Al_2_Ca(SiO_4_)_2_
Anthophyllite	(Mg, Fe^+2^)_7_ Si_8_O_22_
Orthoclase	Al_2_O_3_.K_2_O_6_SiO_2_
Osamilite	K-Na-Ca-Mg-Fe-Al-S
Calcite	CaCO_3_
Vermiculite	22MgO.5Al_2_O_3_.Fe_2_O_3_
Gamet	Ca-Fe-Mg-O-Al-Fe

**Table 3:** BET-adsorption analysis.

Langmuir Data Reduction Parameters					
Adsorbate	Nitrogen Molec. Wt: 28.013	Temperature 77.350 K Cross Section: 16.200	Liquid Density: 0.808 g/cc		
Langmuir					
P/Po	P/Po/W [(g/g)]	P/Po	P/Po/W [(g/g]		

4.94820e-02	2.1679e+00	2.46354e-01	2.9650e+00		
1.20068e-01	2.7030e+00	2.97926e-01	2.9992e+00		
1.83322e-01	2.8789e+00				
Langmuir summary					

	Slope	3.15402			
	Intercept	2.17684			
	Correlation coefficient, r	0.912			
	Surface Area	1104.153 m^2^/g			
Isotherm Data Reduction Parameters					
Adsorbate	Nitrogen Molec. Wt: 28.013	Temperature 77.350 K Cross Section: 16.200	Liquid Density: 0.808 g/cc		
Isotherm Data					
Relative Pressure	Volume @ STP [cc/g]	Relative Pressure	Volume @STP [cc/g]	Relative Pressure	Volume @ STP [cc/g]
4.94820e-02	18.2624	1.83322e-01	50.9498	2.97926e-01	79.4823
1.20068e-01	35.5412	2.46354e-01	66.4800		

DA Method Microspore Analysis Data continued					

Diameter [nm]	dV(d) [cc/nm/g]	Diameter [nm]	dV(d) [cc/nm/g]		

5.54000e+00	2.57659e-02	5.78000e+00	2.22098e-02		
5.56000e+00	2.54455e-02	5.80000e+00	2.19402e-02		
5.58000e+00	2.51296e-02	5.82000e+00	2.16744e-02		
5.60000e+00	2.48184e-02	5.84000e+00	2.14124e-02		
5.62000e+00	2.45115e-02	5.86000e+00	2.11541e-02		
5.64000e+00	2.42091e-02	5.88000e+00	2.08995e-02		
5.66000e+00	2.39110e-02	5.90000e+00	2.06485e-02		
5.68000e+00	2.36172e-02	5.92000e+00	2.04010e-02		
5.70000e+00	2.33276e-02	5.94000e+00	2.01570e-02		
5.72000e+00	2.30421e-02	5.96000e+00	1.99164e-02		
5.74000e+00	2.27606e-02	5.98000e+00	1.96792e-02		
5.76000e+00	2.24832e-02				

### Biodiesel synthesis

#### *Conversion of oil to biodiesel*

Admixture oil (180 mL) was measured and preheated for 30 min in a 500 mL three-necked reactor. 2.5 (wt.) was mixed with 200 mL of ethanol in a separate flask and the mixture was shaken vigorously before being transferred to the preheated oil in the reactor. The observing layers were made uniform using a stirrer and the reaction was monitored for 70 min at a temperature of 70 °C. The products were allowed to set separation layers overnight using a separating funnel. Glycerol was removed from the bottom of the funnel while impure diesel was left in the funnel, and washed with distilled water to remove the ethanol and impurity catalyst left in the biodiesel.

The recycled catalyst was refined, centrifuged, and reused, while the produce biodiesel was made to dryness via calcium chloride. The dried biodiesel was filtered, and computed using Eqn. (2), and kept for further analysis. These steps were repeated until the experimental runs were completed.

(2) $$B_{Y}\left(\%\;wt./wt.\right)=\frac{V_{B}}{V_{AO}}X\;100$$

Where; $B_{Y}$ biodiesel yield, $V_{B}$ volume of the produce biodiesel, $V_{AO}$ volume of the Admixture oil used.

#### *Experimental design by central composite design (CCD)*

The variables, the level and the symbol are displayed in Table 4 as indicated by central composite design (CCD). The reaction temperature was kept constant at 72 °C for complete reaction [7].

**Table 4:** CCD variables level and factors.

Variables	Units	Symbols		Levels			
			−2	−1	0	+1	+2

Reaction time	(min)	X_1_	60	65	70	75	80
Cat. Conc.	(wt.%)	X_2_	2	3	4	5	6
Reaction temp.	( °C)	X_3_	55	60	65	70	75
E-OH/oil molar ratio	(vol. /vol.)	X_4_	4	5	6	7	8

#### *Performance CCD optimization of conversion of admixture oil to biodiesel*

Table 5 shows the experimental yields, the predicted yield, the residual, and the variable's value range. The maximum biodiesel yield of 99.42%(wt/wt) was recorded at a reaction time of 70 min, catalyst conc. of 4 wt.%, reaction temperature of 65 °C, and ethanol/oil molar ratio of 6:1. Table 6a shows the analysis of variance (ANOVA) and test of significant value which indicated the mutual relationship between the variable. The remarkably significant level of linear, interaction, and quadratic terms proved the p-values< 0.001 with high f-values. Table observation indicated that Table 6b indicates the Fits statistical which reflects the level of regression parameter (coefficient of determinations) accounted for model suitability. The mean value serves as the average value of random variables, while the model transfer function that models the system output for each possible input is polynomial.

**Table 5:** Results of experimental values, predicted by CCD, and the residual errors.

**X_1_ (min)**	**X_2_ (%wt.)**	**X_3_. (deg. C)**	**X_4_ (vol. /vol.)**	**BY (% wt. /wt.)**	Pred. yields **(% wt. /wt)**	Res. values
80	4	65	6	94.22	94.33	−0.1071
60	2	75	8	90.73	90.52	0.2092
70	4	65	6	98.65	98.67	−0.0167
70	4	55	6	90.64	90.76	−0.1171
60	2	55	4	90.28	90.25	0.0308
70	4	65	6	98.67	98.67	0.0033
80	6	55	8	95.78	95.83	−0.0538
60	6	75	8	93.10	93.22	−0.1238
60	6	75	4	93.33	93.16	0.1742
80	2	75	8	93.61	93.69	−0.0771
80	6	75	4	93.56	93.58	−0.0171
60	6	55	8	91.20	91.02	0.1825
80	2	55	4	90.00	89.90	0.1046
60	4	65	6	90.15	90.30	−0.1521
70	8	65	6	96.80	96.94	−0.1388
**60**	**2**	**55**	**8**	**87.99**	**87.99**	**−0.0021**
80	2	55	8	90.70	90.60	0.1042
**70**	**4**	**65**	**6**	**99.42**	**99.43**	**0.0033**
60	6	55	4	93.92	93.86	0.0579
70	4	65	6	97.67	97.67	0.0033
60	2	75	4	90.90	90.87	0.0346
70	4	75	6	93.00	93.14	−0.1421
80	6	55	4	94.79	94.72	0.0692
70	2	65	6	89.29	89.41	−0.1204
70	4	65	4	91.00	91.15	−0.1454
70	4	65	6	97.67	97.67	0.0033
80	6	75	8	97.85	97.60	0.2475
70	4	65	6	96.67	96.67	0.0033
80	2	75	4	90.17	90.07	0.0958
70	4	65	8	91.80	91.91	−0.1138

**Table 6:** (a): ANOVA and Test of significant. (b): Fits statistical test.

Source	Sum of squares	df	Mean Square	F-value P-value	
Model	347.52	14	24.82	1003.58	< 0.0001
**Linearterms**					
$X_{1}$	24.30	1	24.30	982.50	< 0.0001
$X_{2}$	85.01	1	85.01	3437.13	< 0.0001
$X_{3}$	8.53	1	8.53	344.97	< 0.0001
$X_{4}$	0.8855	1	0.8855	35.80	< 0.0001
**Interactionterms**					
$X_{1}X_{2}$	1.47	1	1.47	59.44	< 0.0001
$X_{1}X_{3}$	0.1914	1	0.1914	7.74	0.0140
$X_{1}X_{4}$	15.66	1	15.66	633.21	< 0.0001
$X_{2}X_{3}$	1.75	1	1.75	70.71	< 0.0001
$X_{2}X_{4}$	0.1702	1	0.1702	6.88	0.0192
**Quadraticterms**					
$X_{1}^{2}$	8.48	1	8.48	342.96	< 0.0001
$X_{2}^{2}$	69.17	1	69.17	2796.55	< 0.0001
$X_{3}^{2}$	51.71	1	51.71	2090.57	< 0.0001
$X_{4}^{2}$	77.35	1	77.35	3127.18	< 0.0001
**Others**					
Residual	87.32	1	87.32	3530.47	< 0.0001
Lack of Fit	0.3710	15	0.0247	–	0.15
Pure Error	0.3707	10	0.0371	556.01	-
Cor. Total	0.0003	5	0.0001	–	-

(b)					

**Terms**	**RSM-CCD**				
Mean	0.1889				
Sum	93.50				
Standard deviation	0.1037				
Transfer function	Polynomial				
RSME	0.5674				
R- Square	99.95				
R-Square Adjusted	99.75				

**Fig. 5:**
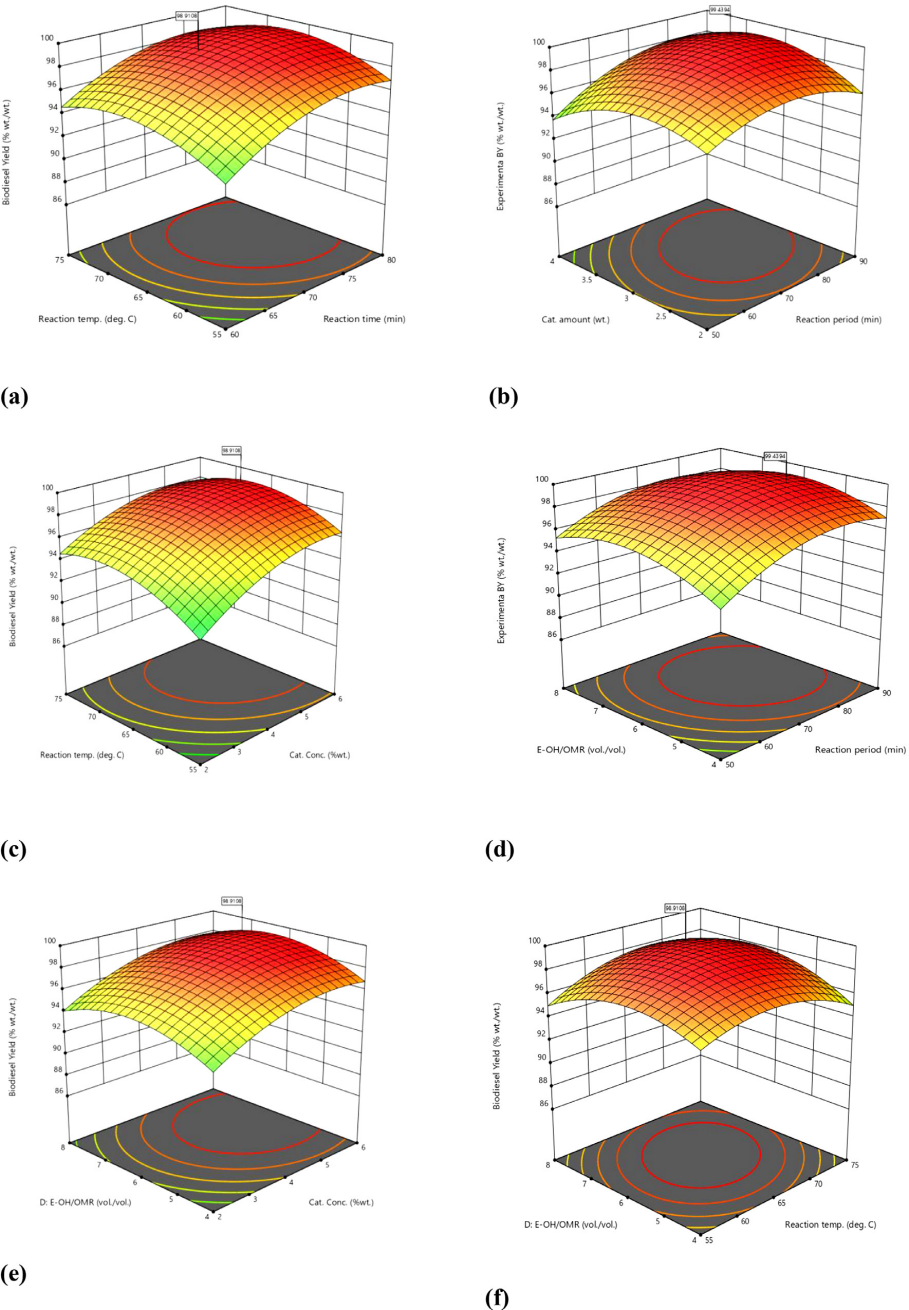
(a-f): Three-dimensional plots.

**Fig. 6:**
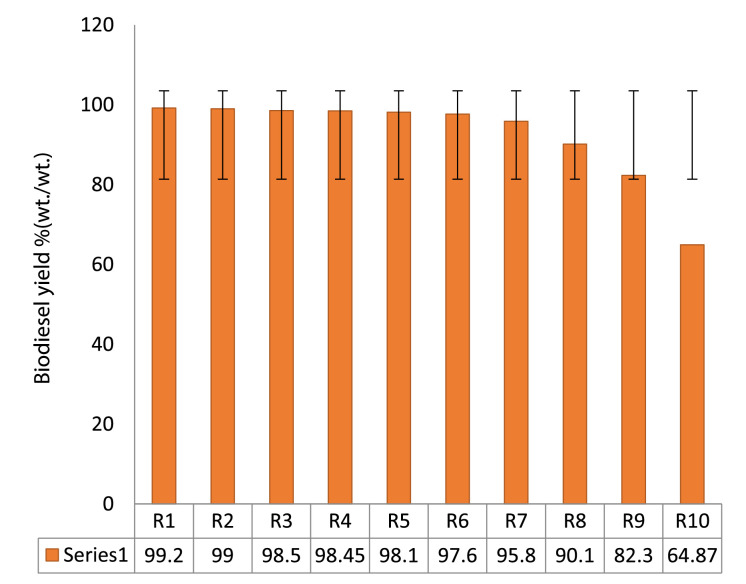
Reusability test plots.

Moreover, the mutual interaction among the variables in terms of XiXj is represented in three-dimensional plots, as displayed in Figs. 5(a-f). Based on the transfer function for (Fig. 6) polynomial order, the model equation describing the optimum output of the yield is presented in Eqn. (3).

**Transfer model equation:**

(3) $$\begin{array}{cc} B_{Y}\%\left(wt./wt.\right) & =347.52+24.30X_{1}+85.01X_{2}+8.53X_{3}+0.89X_{4}++1.47X_{1}X_{2}+0.19X_{1}X_{3}+15.66X_{1}X_{4}+1.75X_{2}X_{3}+0.17X_{2}X_{4}\\ & \;+8.48X_{1}^{2}+69.17X_{2}^{2}+51.71X_{3}^{2}+77.35X_{4}^{2} \end{array}$$

Based on the equation, the predicted yield of 99.43% (wt./wt.), was validated as 99.42%(wt./wt.) at the following conditions: reaction time of 55 min, catalyst conc. of 3.00 (%wt.), reaction temperature of 60 °C, and E-OH/OMR of 5.5:1 (vol./vol.). Hence, the optimum biodiesel yield of 99.42%(wt./wt.) was established.

#### *Physical, chemical and other properties*

The properties (Table 7) of the biodiesel were determined and compared with the biodiesel recommended standard. It was observed that the produced biodiesel properties were in line with the standard recommended values [19,20]. These show that the produced biodiesel can replace conventional diesel with or without blends.

**Table 7:** Properties of the biodiesel.

parameter	biodiesel	[24]	[25]
color@ 27 oc	brownish yellow	–	–
Density (kg/m^3^) @ 25 °C	857	–	860–900
Viscosity @ 40 °C/ (mm^2^/s)	1.86	1.9–6.0	3.5–5.0
Moisture content (%)	<0.001	<0.03	0.02
%FFA (as oleic acid)	0.12	0.40 max	0.25 max
Acid value (mg KOH/g oil)	0.24	0.80 max	0.5 max
Iodine value (g I_2_/100 g oil)	61.00	–	120 max
Saponification value (mg KOH/g oil)	161.10	–	–
Peroxide value (meq O_2_/kg oil)	5.60	–	12.85
HHV (MJ/kg)	41.64	–	–
Cetane number	64.84	57 min	51 min
API	32.03	39.95 max	–
Nature	Light diesel		

#### *Recyclability and reusability test*

The recycled catalyst was refined and reused to examine the catalyst strength. Catalyst recyclability and reusability was tested up to 10 cycles. It was observed that the yield of catalyst reduced drastically at run 6, 7, 8, 9, and 10, respectively, hence the reusability test stop (Fig. 7). The result indicated that the derived heterogeneous catalyst could serve as alkali source for industrial applications.

## Ethics statement

The work does not involve the use of animal or human object.

## Declaration of generative AI and AI-assisted technologies in the writing process

During the preparation of this work the author(s) used the service of Lab. analysis in order to analyzed catalyst sample. After using this service, the author(s) reviewed and edited the content as needed and take(s) full responsibility for the content of the publication.

## CRediT authorship contribution statement

**E.R. Akhabue:** Conceptualization, Methodology, Software, Validation, Formal analysis, Investigation. **S.E. Onoji:** Writing – original draft, Formal analysis, Investigation, Supervision, Methodology. **F. Ishola:** Supervision, Methodology, Software, Validation, Formal analysis, Methodology. **A.A. Ukpong:** Writing – original draft, Investigation, Resources. **O. Idama:** Formal analysis, Resources, Data curation, Writing – original draft. **U. Ekanem:** Data curation, Methodology, Software, Resources, Validation. **T.F. Adepoju:** Writing – original draft, Formal analysis, Investigation.

## Declaration of Competing Interest

The authors declare that they have no known competing financial interests or personal relationships that could have appeared to influence the work reported in this paper.

## Data Availability

No data was used for the research described in the article.
